# Investigation of the role of endosomal Toll-like receptors in murine collagen-induced arthritis reveals a potential role for TLR7 in disease maintenance

**DOI:** 10.1186/ar3875

**Published:** 2012-06-12

**Authors:** Saba Alzabin, Philip Kong, Mino Medghalchi, Andrew Palfreeman, Richard Williams, Sandra Sacre

**Affiliations:** 1 Kennedy Institute of Rheumatology, Nuffield Department of Orthopaedics, Rheumatology and Musculoskeletal Sciences, University of Oxford, London, W6 8LH, UK; 2 Brighton and Sussex Medical School, Trafford Centre, University of Sussex, Brighton, East Sussex, BN1 9RY, UK

## Abstract

**Introduction:**

Endosomal toll-like receptors (TLRs) have recently emerged as potential contributors to the inflammation observed in human and rodent models of rheumatoid arthritis (RA). This study aims to evaluate the role of endosomal TLRs and in particular TLR7 in the murine collagen induced arthritis (CIA) model.

**Methods:**

CIA was induced by injection of collagen in complete Freund's adjuvant. To investigate the effect of endosomal TLRs in the CIA model, mianserin was administered daily from the day of disease onset. The specific role of TLR7 was examined by inducing CIA in TLR7-deficient mice. Disease progression was assessed by measuring clinical score, paw swelling, serum anti-collagen antibodies histological parameters, cytokine production and the percentage of T regulatory (T_reg_) cells.

**Results:**

Therapeutic administration of mianserin to arthritic animals demonstrated a highly protective effect on paw swelling and joint destruction. TLR7^-/- ^mice developed a mild arthritis, where the clinical score and paw swelling were significantly compromised in comparison to the control group. The amelioration of arthritis by mianserin and TLR7 deficiency both corresponded with a reduction in IL-17 responses, histological and clinical scores, and paw swelling.

**Conclusions:**

These data highlight the potential role for endosomal TLRs in the maintenance of inflammation in RA and support the concept of a role for TLR7 in experimental arthritis models. This study also illustrates the potential benefit that may be afforded by therapeutically inhibiting the endosomal TLRs in RA.

## Introduction

Rheumatoid arthritis (RA) is a chronic autoimmune inflammatory disease that affects 1% of the population. Disease progression is characterized by a destructive inflammation of the joints, which can lead to progressive disability and a reduced life expectancy. The synovial membrane in RA is infiltrated by activated immune cells, most abundantly macrophages and T cells, resulting in the chronic production of pro-inflammatory cytokines and matrix metalloproteinases, leading to inflammation and cartilage and bone degradation [1]. The treatment of RA has been revolutionized by the development of biological therapies specifically targeting immune mediators. These include tumor necrosis factor (TNF) [2], interleukin-1 (IL-1), the IL-6 receptor, B cells, and activated T cells (reviewed in [3]). However, these biologics are not orally available and are expensive to manufacture; their cost severely limits use. Side effects are also common; for example, systemic inhibition of TNF confers an increased risk of infection in patients [4]. Thus, there is a requirement for cheaper and more targeted therapies to treat RA.

To improve the therapies available to patients, it is essential to gain a better understanding of the mechanisms that sustain inflammation in RA. Despite the effectiveness of biological therapies in many patients, disease activity usually resumes once treatment has stopped. This indicates that the upstream mechanisms that generate inflammation are still functional and most likely unaffected by these treatments. Many studies from both murine and human models have suggested a role for a family of innate immune receptors, the Toll-like receptors (TLRs) in RA pathogenesis [5].

TLRs form part of a network of receptors that alert the host to the presence of infection and tissue damage. TLRs can be classified into two distinct groups on the basis of cellular distribution and ligand repertoire. Cell surface-expressed TLRs 1, 2, 4, 5, and 6 recognize ligands of mainly bacterial and fungal origin, whereas TLRs 3, 7, 8, and 9 are expressed intracellularly in endosomes and detect nucleic acids from bacteria and viruses [6]. TLR activation induces a strong inflammatory response, which is characterized by the increased expression of TNF among many other mediators. In addition to pathogen-associated ligands, TLRs can engage a number of endogenous molecules that can be produced during tissue damage and are often found at the sites of chronic inflammation [7].

The concept of endogenous ligand-driven activation of TLRs makes these receptors potential candidates for the induction or maintenance (or both) of chronic inflammatory conditions [8]. A potential role has been emerging for the endosomal TLRs in autoimmune diseases such as RA and systemic lupus erythematosus (SLE), in which it has become apparent that DNA- and RNA-associated autoantigen immune complexes can activate B cells and dendritic cells through activation of TLRs [9-11]. Clinical data supporting this concept have come from the study of patients deficient in Unc93B1 [12], a protein required for TLR3, 7, 8, and 9 signaling [13]. Unc93b1-deficient patients show increased numbers of naïve autoreactive B cells in the periphery, similar to patients with RA [12], but do not develop autoreactive antibodies or autoimmunity [12].

In a previous study, we identified an off-target effect of the antidepressant mianserin and showed that it inhibited the activation of the endosomal TLRs 3, 7, 8, and 9 and significantly decreased TNF and IL-6 production from human RA synovial membrane cultures [14]. In the present study, we set out to investigate the role of the endosomal TLRs *in vivo *in an experimental arthritis model using mianserin. Previous work had suggested that TLR8 may be of importance in a human model of RA [14]. However, with no defined ligand, the role of murine TLR8 remains unclear. One study suggests that murine TLR8 may not be functional [15] -leaving murine TLR7 more closely mirroring the behavior of human TLR8. Murine TLR7 and human TLR8 are activated by the same ligands [15-17] and induce TNF production from macrophages, a key mediator of the disease process in RA [18]. Thus, in this study, we chose to focus on the role of TLR7 in the murine CIA model using TLR7^-/- ^mice.

## Materials and methods

### Reagents

Cell culture reagents used were penicillin/streptomycin, Roswell Park Memorial Institute (RPMI) 1640, and Dulbecco's modified Eagle's medium (DMEM) obtained from Cambrex (Verviers, Belgium); fetal bovine serum (FBS) from PAA (Pasching, Austria); and tissue culture-grade beta mercaptoethanol (2-ME) from Gibco-Invitrogen (Paisley, UK). The TLR ligands used were chloroform-extracted, *Escherichia coli *lipopolysaccharide (LPS), resiquimod (R-848), and CpG (ODN M326) from InvivoGen (San Diego, CA, USA). Mianserin hydrochloride was purchased from Sequoia Research Products (Pangbourne, UK). All reagents were tested for LPS contamination by using the limulus amebocyte lysate (LAL) assay from Cambrex (Charles City, IA, USA) [19] and were found to be below 10 pg/mL. Macrophage colony-stimulating factor (M-CSF) was purchased from PeproTech (London, UK).

### Cell culture

Murine bone marrow-derived macrophages (BMMs) were obtained from femurs of male C57BL/6 mice and were cultured for 6 days with DMEM containing FBS (20% vol/vol), 100 U/mL penicillin/streptomycin, 2-ME (50 μM), and 10 ng/mL M-CSF. Draining lymph node cells (DLNCs) were isolated from the inguinal lymph nodes. Cells were cultured in 96-well plates at a density of 2 × 10^5 ^per well in RPMI 1640 containing 10% heat-inactivated fetal calf serum (vol/vol), 100 U/mL penicillin/streptomycin, 2-ME (50 μM), and 1% L-glutamine. Cells were cultured alone or in the presence of 50 μg/mL type II collagen or 100 ng/mL anti-CD3 monoclonal antibody. Supernatants were collected after 48 hours for the determination of IL-17 and interferon-gamma (IFNγ). Cell viability was not significantly affected over this time period when examined by the 3-[4,5 dimethylthiazol-2-yl]-2,5-diphenyl-tetrazolium bromide (MTT) assay (Sigma-Aldrich, St. Louis, MO, USA) [20].

### Cytokine enzyme-linked immunosorbent assay

Sandwich enzyme-linked immunosorbent assays (ELISAs) were employed to measure RANTES (Regulated upon Activation, Normal T-cell Expressed, and Secreted) (R&D Systems, Abingdon, UK) and TNF, IFNγ, and IL-17 (Becton Dickinson, Oxford, UK) in accordance with the instructions of the manufacturer. Absorbance was read on a spectrophotometric ELISA plate reader (Multiscan Biochromic; Thermo Labsystems, Cambridge, UK) and analyzed by using Ascent software version 2.6 (Thermo Labsystems, Cambridge, UK).

### Collagen-induced arthritis

CIA was induced and the clinical score was assessed daily as previously described [21,22] in three independent experiments for both the mianserin and the TLR7^-/- ^experiments. DBA/1 and C57BL/6 mice were purchased and housed in the same unit under conditions identical to those of the TLR7^-/- ^animals. Briefly, 8- to 12-week-old male DBA/1 or C57BL/6 wild-type (WT) or TLR7^-/- ^mice were immunized subcutaneously at the base of the tail with 200 μg of bovine or chicken type II collagen, respectively, emulsified in complete Freund's adjuvant (CFA) (Difco Laboratories, West Molesey, UK). Mianserin treatment was administered therapeutically, starting on the day of onset of arthritis symptoms by interperitoneal injection daily for 7 days in DBA/1 mice. Paw swelling was assessed daily by measuring hind paw thickness by means of calipers. The onset of arthritis was considered to be the day that erythema or swelling (or both) were first observed, and arthritic mice were given a clinical score per limb from 0 to 3, with 0 = normal, 1 = slight erythema or swelling (or both), 2 = pronounced edematous swelling, and 3 = joint deformity with ankylosis, resulting in a maximum score of 12 per animal. This research was approved by the Ethical Review Process Committee of the Kennedy Institute of Rheumatology and the UK Home Office (PPL 70/6533).

### Measurement of IgG1 and IgG2a/c antibodies

Anti-CII IgG levels were measured in mouse sera as previously described [23] with modifications. Briefly, microtiter plates were coated with 2 μg/mL of CII dissolved in 0.05 M Tris-HCl and 0.2 M NaCl (pH 7.4) overnight at 4°C. After blocking for 1 hour with 2% bovine serum albumin, sera were titrated in parallel to a standard sample. A standard consisting of pooled sera was used for the TLR7^-/- ^experiments. For isotype quantitation, sheep anti-mouse IgG1 and IgG2a/c (BD Pharmingen, San Diego, CA, USA) linked to horseradish peroxidase were used at a dilution of 1:1,000. The plates were developed by using tetramethyl benzidine as the substrate, and optical density was measured at 450 nm. Data were presented as arbitrary units.

### Histological assessment of arthritis

On completion of the experiment, the first limb to show evidence of arthritis was processed for histology. The limb was fixed, decalcified, and embedded before sectioning and staining with hemotoxylin and eosin. Histopathological severity was scored in the tarsometatarsal, metatarsophalangeal, and interphalangeal joints by microscopy in a blinded fashion. The histological severity of arthritis was graded as follows: 0 = normal; 1 = minimal synovitis, cartilage loss, and bone erosions limited to discrete foci; 2 = synovitis and erosions present but normal joint architecture intact; and 3 = synovitis and extensive erosions present and joint architecture disrupted. The data are shown as the average score from the three joints for each mouse.

### Flow cytometry

DLNCs were incubated with CD4-conjugated antibody (BD Pharmingen) for 30 minutes at 4°C. Unstimulated cells were stained with FoxP3 antibody (eBioscience, Hatfield, UK) in accordance with the instructions of the manufacturer. Cells were acquired and analyzed on FACS Canto II by using FACSDIVA software (BD Pharmingen).

### Real-time polymerase chain reaction

Comparative mRNA levels of cytokines were assessed in the joints of mice. RNA was extracted by using the RNAeasy mini kit (Qiagen, Crawley, UK) in accordance with the instructions of the manufacturer. Generation of cDNA was carried by using the ABI High-capacity reverse transcription system with random hexamer primers in accordance with the protocol of the manufacturer (Applied Biosystems Inc., Warrington, UK). Amplification of cDNA was performed on an ABI AB7900HT real-time polymerase chain reaction machine in a 384-well plate by using TaqMan Gene Expression Assays sets from the ABI inventoried library for all genes and mouse hypoxanthine phosphoribosyl-transferase (HPRT) control. The relative concentration of each gene of interest was calculated by using the (ΔΔCt) method [24] and expressed as relative units by using a WT arthritic mouse as a calibrator after normalizing against HPRT.

### Statistical methods

Mean, standard deviation (SD), standard error of the mean (SEM), and statistical significance were calculated by using GraphPad version 3 (GraphPad Software Inc., La Jolla, CA, USA). For statistical analysis, a one-tailed *t *test of paired data was used with a 95% confidence interval. SEM was used for pooled experimental data, whereas SD was used in graphs showing representative experiments. A one-tailed Mann-Whitney test was used with a 95% confidence interval for the CIA data (***P < 0.001, **P < 0.01, and *P < 0.05).

## Results

### Mianserin inhibits cytokine production induced by TLR3, 7, and 9 from murine bone marrow-derived macrophages

**Figure 1:**
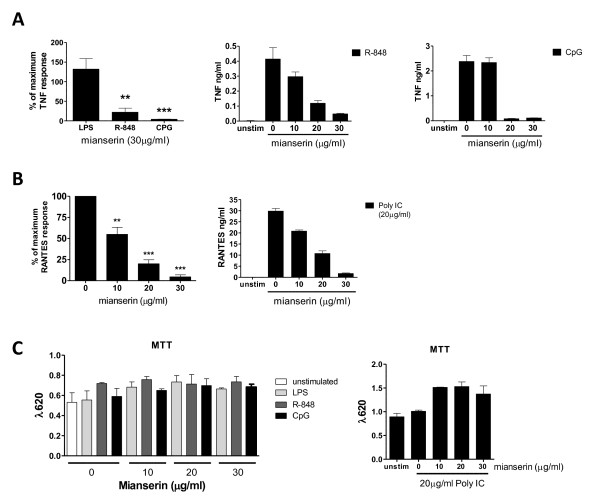
**Mianserin inhibits Toll-like receptor (TLR) 3, 7, and 9 signaling in murine macrophages**. Murine bone marrow-derived macrophages were pre-incubated for 30 minutes with mianserin and then stimulated with **(a) **100 ng/mL lipopolysaccharide (LPS), 1 μg/mL resiquimod (R-848), and 500 μM ODN M326 for 6 hours before measuring tumor necrosis factor (TNF) in the supernantants or **(b) **20 μg/mL polyinosinic:polycytidylic acid (poly I:C) for 24 hours before measuring RANTES production in the supernantants. Data are shown as a percentage of the TLR ligand-only response at 30 μg/mL mianserin and as a representative graph showing the effect of a range of concentrations of mianserin. **(c) **Cell viability was measured by 3-[4,5 dimethylthiazol-2-yl]-2,5-diphenyl-tetrazolium bromide (MTT) assay. **P < 0.01, ***P < 0.001. RANTES, Regulated upon Activation, Normal T-cell Expressed, and Secreted; unstim, unstimulated.

**Figure 2:**
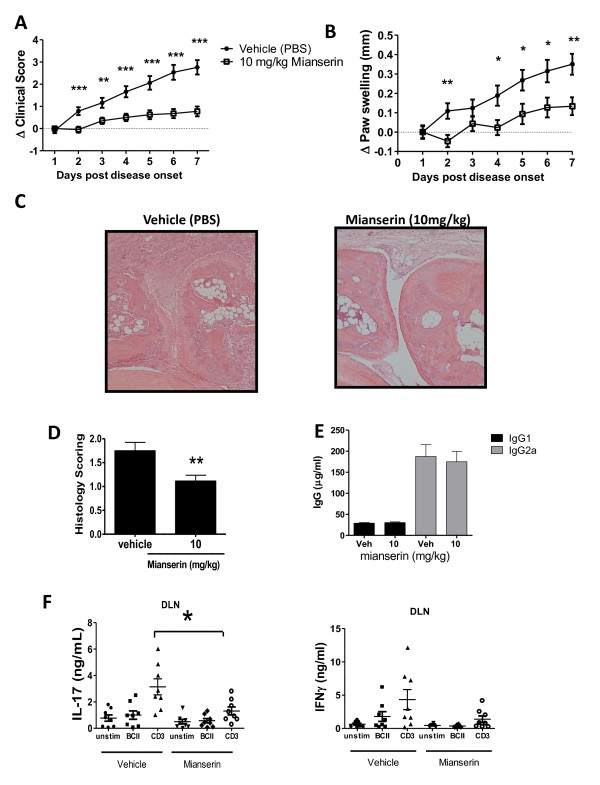
**Mianserin inhibits disease progression, paw swelling, and interleukin-17 (IL-17) production in the collagen-induced arthritis (CIA) model of arthritis**. DBA/1 CIA mice were therapeutically given an intraperitoneal injection of vehicle (phosphate-buffered saline,) or 10 mg/kg mianserin once a day for 7 days, starting on the day of disease onset. Mice were assessed for **(a) **clinical score (*n *= 20 mice per group) and **(b) **paw swelling on a daily basis (*n *= 15 to 17 mice per group). **(c, d) **Arthritis was scored histologically as described in Materials and methods for the vehicle control group (*n *= 18) and the mianserin-treated group (*n *= 17). **(e) **Mice were bled on day 7, and serum levels of IgG_1 _and IgG_2a _anti-collagen antibodies were measured (*n *= 20). Frames (a-e) show data pooled from three separate experiments. **(f) **On day 10 after disease onset, cells from draining inguinal lymph nodes were cultured and stimulated with bovine type II collagen (BCII) (50 mg/mL) or anti-CD3 monoclonal antibody (100 ng/mL) for 48 hours. Supernatants were analyzed for the production of interferon-gamma (IFNγ) and IL-17 by enzyme-linked immunosorbent assay. Each point represents an individual animal (*n *= 8). *P < 0.05, **P < 0.01, ***P < 0.001. unstim, unstimulated.

In a previous study, we demonstrated that mianserin could inhibit the activation of TLRs 3, 7, 8, and 9 but not the cell-surface TLRs 1/2, 4, and 5 in primary human cells [14]. Before testing the effects of mianserin in CIA, it was essential to confirm that mianserin was able to inhibit endosomal TLR activation in murine primary macrophages (BMMs). Mianserin inhibited the production of TNF upon activation of TLR7 and 9, but not TLR4, in BMMs (Figure 1a). RANTES was used as a readout for TLR3 activation because polyinosinic:polycytidylic acid (poly I:C) is a weak inducer of TNF in BMMs. Upon TLR3 stimulation, mianserin also dose-dependently inhibited the production of RANTES (Figure 2b). TLR8 activation was not measured, as the mechanism of activation of this receptor remains unclear at present. Human TLR7 and 8 can be activated by singled-stranded RNA (ssRNA) or resiquimod, but in murine cells only TLR7 (not TLR8) responds to these ligands [15,17]. Cell viability was not affected by mianserin as assessed by the MTT assay (Sigma-Aldrich) [20] (data not shown).

### Mianserin suppresses disease progression in CIA

At the same concentration as was previously shown for inhibition of human TLR signaling, mianserin inhibited the spontaneous production of TNF and IL-6 from human synovial membrane cultures [14]. To investigate whether the beneficial effects of mianserin would also be observed *in vivo*, the murine CIA model was chosen, as it shows many similarities to human RA, such as a comparable synovitis, bone erosion, and pannus formation [25]. Mice were treated with mianserin by using a therapeutic rather than a prophylactic protocol. Mianserin was able to dramatically reduce the clinical score (Figure 2a) and gave a significant reduction in paw swelling (Figure 2b). Increasing the dose to 25 mg/kg gave no added benefit (data not shown). Histological analysis of hind paws in mice treated for 7 days after onset showed that mianserin had a beneficial effect on preserving joint architecture and decreasing both bone destruction and cell infiltration into the joint (Figure 2c). Pooled data from all mice clearly showed the significant effect of mianserin on the histological score (Figure 2d). As would be expected of an intervention given therapeutically, mianserin had no effect on serum levels of anti-collagen IgG1 or IgG2a (Figure 2e).

IL-17-producing CD4^+ ^T cells have been shown to be important contributors to pathogenesis in the CIA model [26]. Thus, the decreased disease progression observed in the mianserin-treated group might be due to reduced numbers of pathogenic T cells. To test this hypothesis, DLNCs from the mice treated with mianserin showed a significant decrease in anti-CD3-induced IL-17 production; a strong trend toward lower production of antigen-specific IFNγ and IL-17 was observed, although this did not reach statistical significance (Figure 2f). In contrast, the total numbers of CD4^+ ^cells and the percentage of CD4^+^foxp3^+ ^cells were unchanged in the DLNs of mianserin-treated mice (data not shown).

### TLR7 is required for the maintenance of disease in CIA

The ability of mianserin to inhibit endosomal TLR activity in BMM and effectively suppress disease progression in CIA raises the possibility that the anti-inflammatory activity of mianserin is due to its ability to dampen one or more of the endosomal TLR responses. Data from the human RA model previously suggested that TLR8 was a key contributor to cytokine production from human synovial membrane tissue [14]. However, since the function of murine TLR8 is unclear at present [17,27], its paralogue murine TLR7 (which more closely reflects the function of human TLR8) was investigated by using TLR7^-/- ^mice.

**Figure 3:**
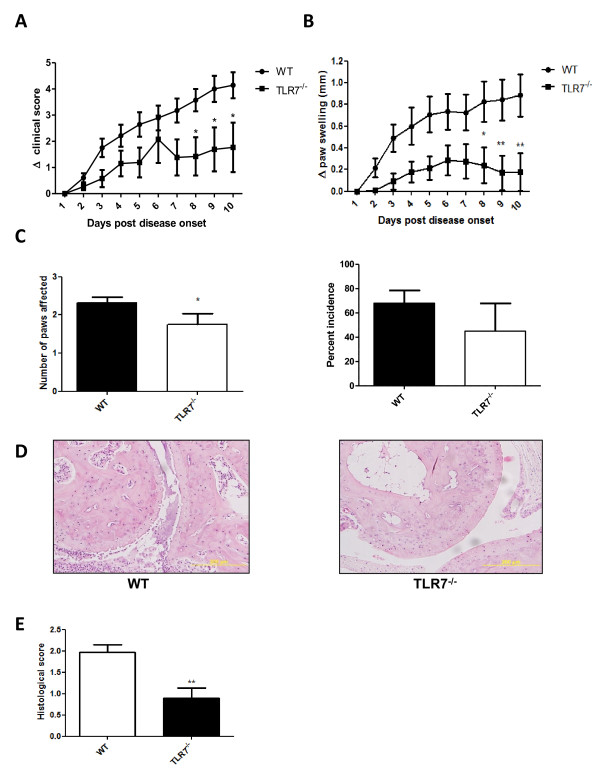
**Suppression of arthritis in TLR7-deficient mice**. TLR7^-/- ^(*n *= 12) and control C57Bl/6 (*n *= 14) mice were immunized with chicken collagen in complete Freund's adjuvant. Arthritis was assessed by **(a) **clinical score and **(b) **paw swelling on a daily basis from the first day of disease onset. Data are pooled from three independent experiments. **(c) **The number of paws affected and the disease incidence were recorded for each group from three separate experiments. **(d) **The first affected joint was collected after 10 days of disease onset and was fixed and stained with hematoxylin and eosin. **(e) **Arthritis was scored histologically for TLR7^-/- ^(*n *= 9) and control C57Bl/6 (*n *= 10) mice as described in Materials and methods and pooled from two independent experiments. *P < 0.05, **P < 0.01. TLR, Toll-like receptor; WT, wild-type.

CIA was induced in TLR7^-/- ^and control C57BL/6 mice and disease was assessed as described above. Clinical features were measured only in mice exhibiting clinical signs of arthritis. TLR7 deficiency significantly compromised the clinical score, progression in paw swelling, and number of paws affected (Figure 3a-c, respectively). The percentage incidence was also decreased but this decrease did not reach statistical significance (Figure 3c). Histological analysis of the affected joints from arthritic TLR7^-/- ^mice at day 10 after onset clearly showed a reduction in disease parameters with minimal inflammation and synovitis and a clear preservation of the joint architecture (Figure 3d, e).

### Protection from CIA progression in TLR7-deficient mice is associated with decreased antigen-specific IL-17 production and increased T_reg _cells

**Figure 4:**
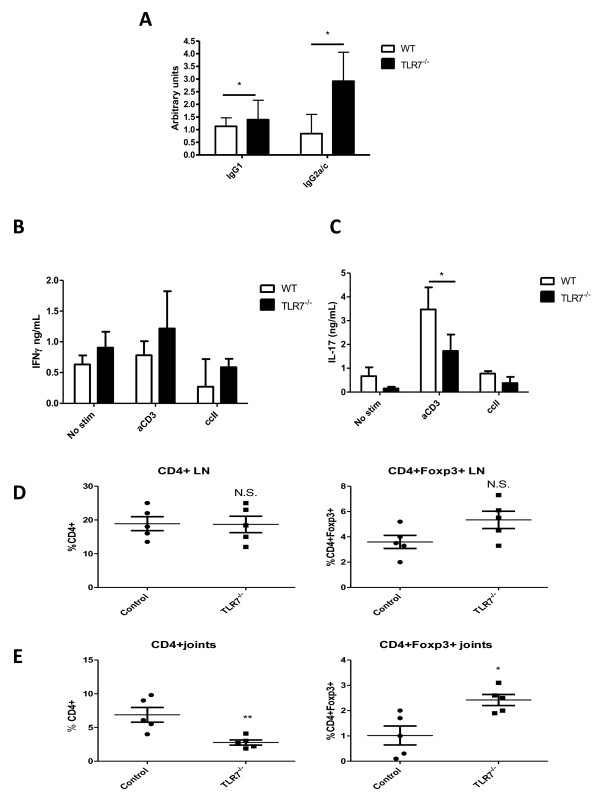
**Arthritis in TLR7^-/- ^mice is associated with decreased IL-17 levels and increased T_reg _cells**. **(a) **Mice from Figure 3 were bled on day 10, and serum levels of IgG_1 _and IgG_2a/c _anti-collagen antibodies were measured by enzyme-linked immunosorbent assay as described in Materials and methods. **(b, c) **Draining lymph node cells (DLNCs) were isolated 10 days after disease onset and stimulated with 100 ng/mL anti-CD3 monoclonal antibody or 50 μg/mL chicken collagen, and supernatants were collected for the measurement of IL-17 (b) and interferon-gamma (IFNγ) (c). **(d) **DLNCs or cells liberated from the affected joints **(e) **were stained with conjugated antibodies to CD4 and foxp3 to determine the percentage of T_reg _cells. *P < 0.05, **P < 0.01. IL-17, interleukin-17; LN, lymph node; No stim, no stimulation; N.S., not significant; TLR, Toll-like receptor; T_reg_, T regulatory; WT, wild-type.

We questioned whether the reduced arthritis observed in TLR7^-/- ^animals was due to a failure to elicit a robust immune response to *Mycobacterium tuberculosis *(MTB), the active ingredient in the adjuvant used in the CIA model. TLR7 deficiency did not affect the innate immune response of BMMs to MTB (data not shown), suggesting an adjuvant-independent regulatory mechanism. Furthermore, TLR7^-/- ^mice actually mounted a stronger B-cell response to type II collagen (which is essential for disease induction in CIA) compared with their WT counterparts, indicating that the humoral arm of the immune response in TLR7^-/- ^mice was not deficient (Figure 4a).

**Figure 5:**
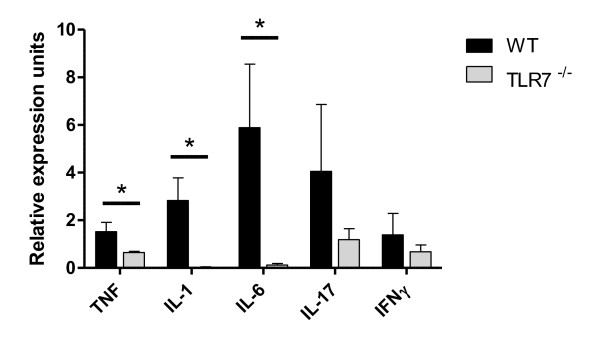
**TLR7^-/- ^mice have decreased cytokine expression in the paw tissue**. RNA was isolated from whole affected paws of control C57Bl/6 wild-type (WT) (*n *= 4 or 5) or TLR7^-/- ^(*n *= 3 or 4) mice. Real-time polymerase chain reaction was performed to measure levels of tumor necrosis factor-alpha (TNFα), interleukin-1-beta (IL-1β), IL-6, IL-17, and interferon-gamma (IFNγ) using ABI primers. *P < 0.05. TLR, Toll-like receptor.

Supporting the reduction in disease parameters, DLNCs from TLR7^-/- ^mice produced significantly less antigen-specific IL-17 in response to collagen stimulation *ex vivo *(Figure 4b), indicating that TLR7 may be involved in regulating T helper 17 (T_H_17) responses. Interestingly, a trend toward increased IFNγ production was observed but was not significant (Figure 4c). Subsequently, the involvement of TLR7^-/- ^T cells was investigated. The overall percentage of CD4^+ ^T cells was markedly reduced in the joints but not the DLNs of TLR7^-/- ^mice compared with WT mice (Figure 4d, e), reflecting a reduced level of inflammatory cell infiltration in the absence of signaling via TLR7. This was supported by a reduction in the mRNA levels of TNFα, IL-1, IL-6, IL-17, and IFNγ (Figure 5) and a decrease in T_H_17 cells in the joints of TLR7^-/- ^when compared with controls (data not shown). In contrast, the percentage of CD4^+^foxp3^+ ^cells was significantly higher in the joints of TLR7^-/- ^mice compared with WT controls, despite the fact thatthe total percentage of CD4^+ ^cells was significantly lower in TLR7^-/- ^animals (Figure 4e). A trend toward increased regulatory T (T_reg_) cells in the DLNs of TLR7^-/- ^animals was also observed but did not reach significance (Figure 4d).

## Discussion

In this study, we set out to investigate the role of endosomal TLRs in a murine model of RA by using mianserin, an antidepressant that can inhibit endosomal TLR signaling [14]. Therapeutic administration of mianserin decreased disease progression and appreciably preserved the joint architecture in the CIA model, suggesting a possible role for one or more of these TLRs in the maintenance of disease. This result was consistent with previous data, in which we showed that mianserin could inhibit spontaneous production of TNF and IL-6 from human RA synovial membrane cultures [14]. Conversely, some reports have suggested that 5HT-2A receptor antagonists, including mianserin, can cause adverse drug reactions, including joint problems [28,29]. However, the discrepancy between these reports and our studies may be explained by the fact that the inhibition of TLRs by mianserin is an off-target effect observed only at doses that are higher than would be safe to administer clinically [30]. Thus, mianserin may produce differing effects via distinct mechanisms when used at low or high concentrations. Accordingly, mianserin does not represent a useful anti-arthritic drug for the clinic. However, these data highlight the potential benefit that might be provided by the development of a more specific set of inhibitors of the endosomal TLRs.

Previous data from a human model of RA had suggested a role for TLR8 in the production of TNF [14]; however, extrapolating this finding across species becomes difficult when considering the role of TLR8. Whereas human TLR7 and 8 can be activated by ssRNA or resiquimod, only TLR7 (and not TLR8) responds to these ligands in rodents [15,17]. This distinction was recently suggested to be due to a variation between species in leucine-rich repeats 14-15 of TLR8 [31]. The mechanism of murine TLR8 activation and the function of this receptor remain unclear at present. Nonetheless, its functional paralogue murine TLR7 behaves in a manner similar to human TLR8; both receptors are known to be activated by the same natural ligand, ssRNA, and can induce TNF production from macrophages [17]. Thus, this study focused on the role of TLR7, rather than TLR8, in the CIA model.

Here, we show that TLR7 deficiency resulted in a decrease in clinical and histological scores, paw swelling, and disease incidence. Compared with control WT mice in which the clinical score progresses steadily after disease onset, the clinical score in the majority of TLR7^-/- ^mice failed to increase. Interestingly, disease resolved in about 20% of arthritic mice within 5 days of onset. Initiation of CIA requires CFA, which is a mixture of mineral oil and heat-inactivated MTB; thus, it was possible that TLR7 deficiency may haveprevented the induction of arthritis. Encouragingly, both BMMs and dendritic cells from TLR7-deficient mice responded to heat-inactivated MTB, producing TNF levels comparable to those of cells from WT mice (data not shown). This suggested that the innate immune response to MTB remained intact in TLR7-deficient mice.

Another important mechanism in the initiation of CIA is the generation of anti-collagen antibodies; these antibodies have also been shown to be capable of transferring disease to immunocompromised animals [32,33]. In a murine model of spontaneous lupus, it was shown that TLR7 deficiency results in decreased serum levels of IgG_2a _and IgG3 isotypes [34], which are among the pathogenic isotypes in autoimmune diseases such as SLE [35]. However, it was of interest that we observed that the lack of TLR7 resulted in higher levels of anti-collagen IgG_1 _as well as IgG_2a/c _in the serum of arthritic mice when compared with WT controls, suggesting that TLR7 signaling has a negative impact on T_H_1 responses in the CIA model. Why TLR7 deficiency resulted in opposing effects in two animal models is not understood but may be due to the direct injection of antigen or the presence of adjuvant in the CIA model, perhaps implicating antigen presentation in this discrepancy. Indeed, the increased level of the T_H_1-associated IgG_2a/c _isotype may have supported the increase in IFNγ production from stimulated DLNCs. Of the T_H_1 responses, IFNγ in particular has been associated with protection from disease in experimental models of arthritis (reviewed in [36]). One way in which IFNγ suppresses disease is by inhibiting IL-17 production [37]. Likewise, arthritic TLR7^-/- ^animals showed a significant decrease in IL-17/T_H_17 and an increase in T_reg _cells, pointing to a potential role for TLR7 in regulating T-cell plasticity or the balance between T_H_17 cell/T_reg _cell responses or both. However, on the basis of our findings, it was not possible to establish whether TLR7 deficiency has a direct effect on T cells or acts indirectly via the antigen-presenting cells. T_reg _cells have also been implicated in the control of inflammatory arthritis in animal models (reviewed in [36]). TLR7 ligation is reported to suppress foxp3 expression [38] and thus may explain the increase in T_reg _cells in TLR7-deficient arthritic mice. However, no change in the level of T_reg _cells was observed in DLNs of mianserin-treated mice and this may reflect the difference of specific TLR7 deficiency as opposed to pan-endosomal TLR inhibition.

However, our data do not exclude a role for other TLRs. In the rat pristane model of arthritis, recent studies have shown upregulation of TLR3 in the rat synovium, aggravation of arthritis by the TLR3 ligand poly I:C, and amelioration of disease by TLR3 RNA interference *in vivo *[39,40]. TLR9 inhibitors have also been reported to delay disease onset and severity in the rat pristane model [41].

Most studies of murine experimental arthritis have suggested a contribution from TLR4 to the maintenance of inflammation [42-44]. A role for TLR7 may, in fact, be complementary to these studies, as TLR7-induced type I IFN has been shown to enhance TLR4 activation, most notably in cells taken from patients with RA [45]. Involvement of TLR7 in the maintenance of inflammation in CIA is also in keeping with other studies of inflammatory arthritis models. Low-dose activation of TLR7 with a small synthetic ligand has been shown to induce tolerance of TLR2, 7, and 9 signaling and to suppress disease in a serum transfer model of arthritis [46]. In addition, a recent study of pristane-induced arthritis has shown that splenocytes from an arthritic animal can transfer disease after re-stimulation with heterogeneous nuclear ribonucleoprotein (hnRNP) antigens that activate TLR7 and 9 [47]. Intra-articular lentiviral delivery of TLR7 short-hairpin RNA was recently shown to decrease IL-1 and IL-6 expression in synovial tissue of CIA rats [48]. This is in agreement with our data showing a decrease in IL-1, IL-6, and other cytokines in the paw tissue from the TLR7^-/- ^CIA mice.

## Conclusions

In summary, this study has uncovered an important role for TLR7 in CIA which complements other animal model studies [46,47] and shows a role comparable to that of its human homologue, TLR8, in RA [14]. Given that the activity of TLR7 and 8 can be modulated by small synthetic agents such as mianserin, these receptors may provide amenable targets for the development of new therapeutics for RA.

## Abbreviations

2-ME: beta mercaptoethanol; BMM: bone marrow-derived macrophage; CIA: collagen-induced arthritis; DLN: draining lymph node; DLNC: draining lymph node cell; DMEM: Dulbecco's modified Eagle's medium; ELISA: enzyme-linked immunosorbent assay; FBS: fetal bovine serum; HPRT: hypoxanthine phosphoribosyl-transferase; IFN: interferon; IL: interleukin; LPS: lipopolysaccharide; M-CSF: macrophage colony-stimulating factor; MTB: *Mycobacterium tuberculosis*; MTT: 3-[4,5 dimethylthiazol-2-yl]-2,5-diphenyl-tetrazolium bromide; poly I:C: polyinosinic:polycytidylic acid; RA: rheumatoid arthritis; RANTES: Regulated upon Activation, Normal T-cell Expressed, and Secreted; RPMI: Roswell Park Memorial Institute; SD: standard deviation; SEM: standard error of the mean; SLE: systemic lupus erythematosus; ssRNA: single-stranded RNA; T_H_: T helper; TLR: Toll-like receptor; TNF: tumor necrosis factor; T_reg_: T regulatory; WT: wild-type.

## Competing interests

SS has filed a patent application (WO/2008/090334) entitled 'Use of 5HT Receptor Antagonists for Treating Arthritis' and has no financial benefit to declare. The other authors declare that they have no competing interests.

## Authors' contributions

SA participated in the design of the study, performed the TLR7^-/- ^experiments, analyzed the results, and drafted the manuscript. SS participated in the design of the study, carried out the mianserin experiments, analyzed the results, and drafted the manuscript. MM performed the mianserin CIA trials. PK stimulated the DLNCs from the mianserin experiments. AP performed the real-time polymerase chain reaction experiments. RW performed the histology scoring for the mianserin CIA data and participated in the analysis of the results and writing of the manuscript. All authors read and approved the final manuscript.
